# Clinical and electromyographic signals analysis about the effect of space-adjustment splint on overerupted maxillary molars

**DOI:** 10.1186/s12903-024-04039-6

**Published:** 2024-03-02

**Authors:** Qun Lei, Dong Lin, Kaijin Lin, Wenxiu Huang, Dong Wu, Yuyu Liu

**Affiliations:** 1 Fujian Key Laboratory of Oral Diseases & Fujian Provincial Engineering Research Center of Oral Biomaterial & Stomatological Key Laboratory of Fujian College and University, School and Hospital of Stomatology, Fujian Medical University, Fuzhou, China; 2 Fujian Medical University, Fuzhou, China

**Keywords:** Molars, Occlusal splints, Electromyography, Dental implant, Prostheses, Orthodontics

## Abstract

**Background:**

Overerupted maxillary molars is common in adults, which can lead to insufficient intermaxillary vertical space ,great difficulty in prosthetic reconstruction ,and cause occlusal interference in movements.To reconstruct occlusal function, it is necessary to prepare enough space for prostheses. The aim of the present study was to evaluate the effect of space-adjustment occlusal splint on overerupted maxillary molars by clinical and electromyographic signals analysis.

**Methods:**

Eighteen patients with overerupted maxillary molars were selected to wear space-adjustment occlusal splint suppressing overerupted maxillary molars for three months. Satisfaction was assessed by 5-point Likert; intermaxillary vertical space and the teeth transportation distance were measured in models; clinical periodontal status were evaluated by periodontal probing depth (PPT) and bleeding index (BI); electromyographic recordings of the masseter and anterior temporal muscles were monitored by Cranio-Mandibular K7 Evaluation System.

**Results:**

All the patients were satisfied with the treatment effect (Likert scale ≧ 4). The intermaxillary space in edentulous areas after treatment showed statistically significant increasing when compared with those before treatment. PPT and BI showed no significant difference. No statistically significant differences were found in electromyographic activity of anterior temporal muscles, while a reduction of muscle activity in masseter in the contralateral side were detected in post-treatment evaluations compared with pre-treatment at mandibular rest position.

**Conclusions:**

Space-adjustment occlusal splint is an efficient treatment option on overerupted maxillary molars by intruding the maxillary molar to obtain adequate intermaxillary space for prostheses.

## Background

Due to the long-term loss of antagonists and low bone density in the posterior maxillary regions, it is common to see overerupted maxillary molars in adults, which can lead to insufficient intermaxillary vertical space, great difficulty in prosthetic reconstruction [1], cause occlusal interference in protruding, lateral or retreating movements [2],and temporomandibular dysfunction in severe cases [3, 4].

Various approaches have been applied to intrude supraerupted molars, including the application of crown truncation, orthodontic treatment with removable or fixed appliances, subapical osteotomy and surgical extraction. Crown truncation is a common option to resolve overerupted molars in a short time at the expense of pulp vitality. For the normal occlusal curve and adequate space for the restoration of antagonist teeth, it can be satisfied by tooth preparation after endodontic treatment of extruded antagonist molar rather than simple grinding. Orthodontic intrusion with various devices applied is another option in clinical practice [5], such as fixed appliances, removable appliances, extraoral devices, and lately, the use of mini-screws [6–8]. Mini-screws belonging to skeletal anchorage systems can promote the teeth movement immediately after the placement with elastomeric chains. It can significantly reduce the treatment time and intraoral devices. And yet, this method also presents some shortcomings, including mucosal ulcer, mini-screws loss and periodontal problems caused by local devices. In addition, the application of mini-screw is limited under some circumstances. For example, the distance between the roots of two teeth is too close, the amount of alveolar bone in the implanted area is insufficient, and the patient’s mouth is open small. All these factors will limit the mini-screws implanted to the correct position. In addition to the above methods, subapical osteotomy and surgical extraction can be performed to correct the occlusal curve when the antagonist teeth erupt too much and other methods are invalid. However, these treatments need to be completed with more trauma.

Space-adjustment occlusal splint is a removable device, which can retain by clasped in the remained teeth and restore missing teeth. Bite force can be localized exerted on erupted antagonist teeth by splint to create the enough space for prosthetic rehabilitation. Compared with other methods, space-adjustment occlusal splint treatment has the advantages of small trauma, low cost, strong flexibility, high aesthetics and good comforts.

The aim of this study was to investigate the safety and effectiveness of bite force on overerupted maxillary molars through space-adjustment occlusal splint.

## Methods

### Patients

22 patients were included, of which age ranging between 20 and 50 years from School and Hospital of Stomatology, Fujian Medical University. Participants were treated with space-adjustment occlusal splint based on the presence of one or two mandibular molar loss, overerupted maxillary molars and stable occlusal relationship, absence of active periodontal disease and temporomandibular disorder, and absence of bruxism. The splint with recovered mandible molars was designed to act on overerupted maxillary molars. All participants were asked to use the device for at least 12 hours a day beside the time for taking food and brushing your teeth. The subjects received oral hygiene treatment and guidance before the study. If patients could not follow the doctor’ advice, they were excluded from research. This study was approved by Biomedical Research Ethics Review Committee, Stomatological Hospital, Fujian Medical University ([2020] Fuyikou Ethics Review Character No. (34); [2019] Fuyikou Ethics Review Character (36)). All patients provided written informed consent. I confirm that all methods were performed in accordance with the relevant guidelines.

### Space-adjustment occlusal splint

**Fig. 1:**
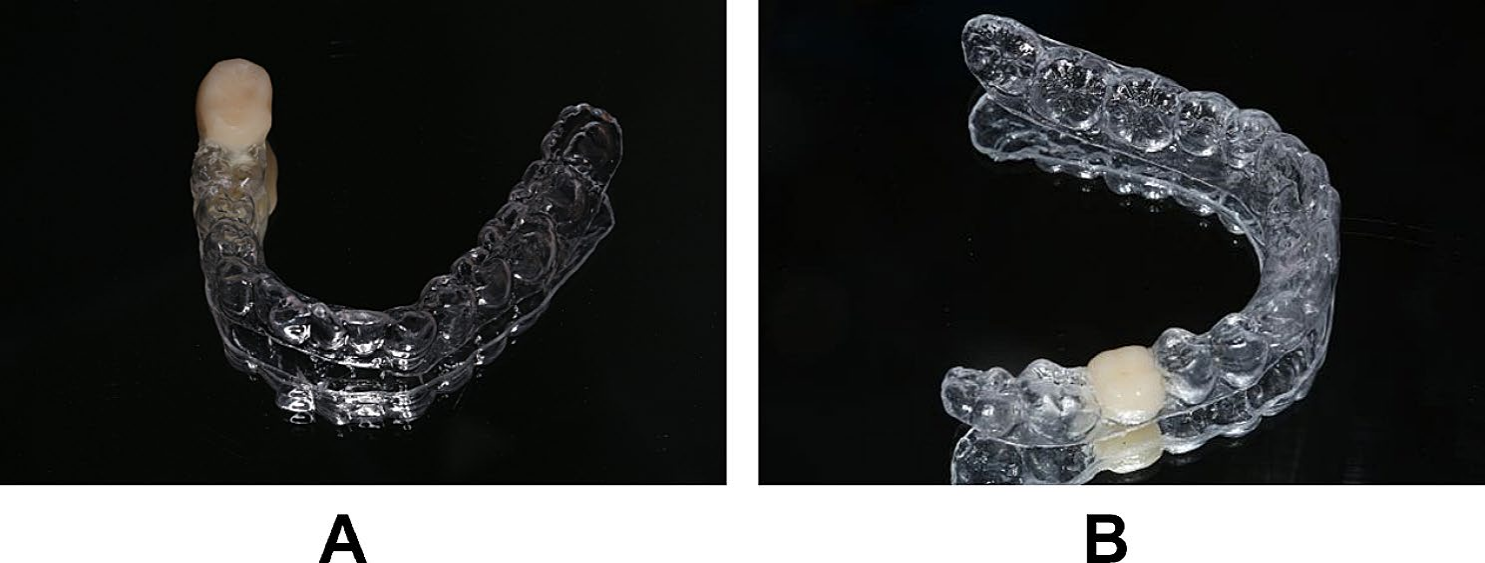
Space-adjustment occlusal splint

Space-adjustment occlusal splint in Fig. 1 was fabricated from a 1 mm polyvinyl sheet and tooth coloured self-curing resin. As the retainer for the splint, sheet was properly adapted to the mandible gypsum dental cast in a vacuum pressure molding device with an infrared heater. Then self-curing resin was fixed on splint to restore the shape and bite function of the lost mandible molar. The occlusal height of the mandible molar was decided according to intermaxillary vertical distance at mandibular rest position (free-way space). The occlusal point between overerupted maxillary molar and mandible molar recovered by self-curing resin was adjusted to ensure that the bite force acted on the obviously elongated cusp first.

### Pain scores (Numerical Rating Scale) and satisfaction evaluation (5-point likert scale)

Patient pain scores and satisfaction survey were used to assess patient experience after undergoing occlusal splint therapy on overerupted maxillary molars. Patient pain severity was evaluated by Numerical Rating Scale (NRS) on a scale from 0 to 10 (rating scale: 0,no pain;1–3,mild pain; 4–6,moderate pain; 7–10,severe pain) [9, 10]and the level of satisfaction about treatment effect was assessed by 5-point Likert scale ranging from “strongly satisfied” to “strongly dissatisfied” (1, strongly dissatisfied; 2, dissatisfied; 3, fair; and 4, satisfied; and 5, strongly satisfied) [11, 12]. Patients were asked to record the NRS every two week during the following 3 month, and the degree of satisfaction with therapeutic effect was assessed at final follow-up.

### Clinical periodontal parameters

Periodontal Probing depth (PPT) from the base of the gingival pocket to the free gingival margin was measured by evaluator using a William periodontal probe marked in millimeters (mm). It was performed at 6 sites of the overerupted teeth (mesiobuccal, buccal, distobuccal, mesiolingual, lingual, distolingual). Bleeding index (BI) was evaluated by observing the presence or absence of bleeding, and the degree of bleeding up to 30 s after finishing probing depth. The score was record on a scale from 0 to 5 according Mazza(1981) method(0, normal appearing, healthy gingiva;1,color changes related to inflammation but no bleeding;2,slight bleeding that remains at the point of sampling; 3, bleeding extending from the point of sampling and flowing around the gingival margin;4,profuse bleeding that overflows the gingival margin;5,spontaneous bleeding [13]. The probing depth was the average of values at 6 sites, while bleeding index recorded was the most severe form of bleeding after probing in 6 sites. Data collections were performed before treatment(T0), 6-weeks (T1) treatment, 12-weeks (T2) treatment, 2-weeks after treatment ended(T3).

### Intermaxillary vertical space and the teeth transportation distance

Study models were made for each participant before treatment, after 6 weeks and 12 weeks treatment. The models were required to have complete dental arch shape, basal bone, and avoid bubbles as much as possible. With the tooth at intercuspal position, shortest distance was measured by vernier caliper in plaster models from overerupted cusp of the maxillary molar to the alveolar crest of the edentulous areas. The data of intermaxillary space calculated in the edentulous areas was the average of three values by 3 different surveyors. The data obtained before treatment(T0) was used as the reference to analyze the spacing change in the edentulous areas after 6-weeks (T1) treatment and 12-weeks (T2) treatment.

**Fig. 2:**
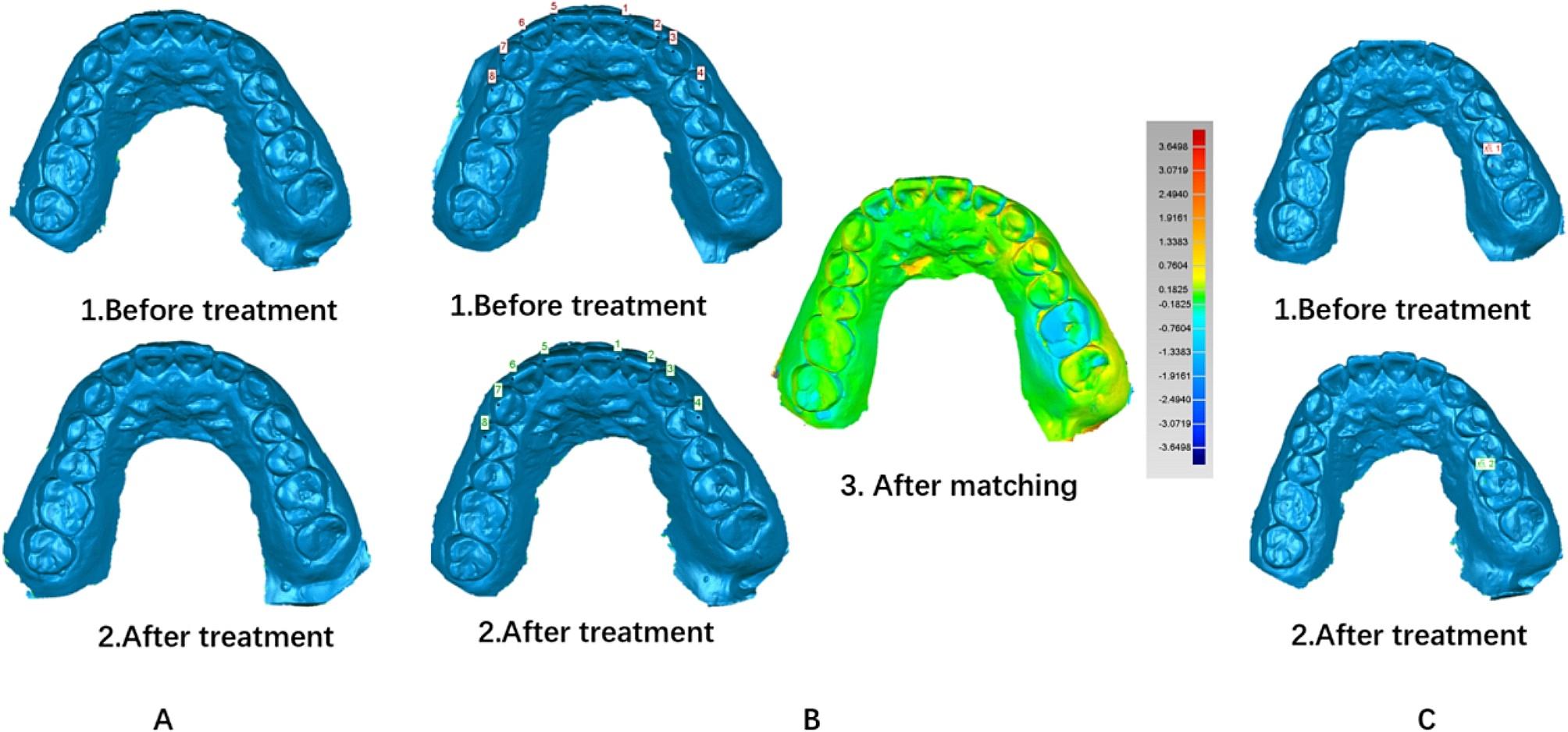
Management of compared digital models **A**, Digital model acquisition: All casts were scanned using a scanner (TRIOS POD 2, 3 Shape Inc.) performed by an experienced operator. All digital models were assessed using reverse engineering software (Geomagic 2013, 3D Systems Inc.). **B**, Digital model matching: “N-point alignment” of two digital models used the selected “characteristic points” as a reference. After aligning the two models to the same coordinate system, the best fitting alignment was carried out, and the number of sampling points was 5000.RMS and color-coded map were calculated to check the alignment. In the spectrum, the color was closer to the center of green, the RMS was less, Distance calculation: After the best fitting alignment, the elongated tip of the target teeth on the digital models were selected and the distance of straight line between the two points was calculated

The maxillary superhard plaster model was then scanned by using 3 Shape scanning chamber according to the manufacturer’s instructions, and the digital model obtained was exported as STL files, and then imported into Geomagic Wrap 2013 software to reconstruct and measure. The “characteristic points” in maxillary except for adjacent tooth were selected as the fitting reference areas, and then the model was fitted using the best fit alignment to obtain the overlapping digital model [14, 15]. Color-coded map was used to check alignment. The model obtained before treatment (T0) was used as the reference model while the others obtained after 6-weeks (T1) treatment and 12-weeks (T2) treatment as test model. The elongated tip was pointed out on the reference model and test model respectively, where the corresponding moving distance was measured in Fig. 2. The data of transportation distance calculated between two points was the average of three values by 3 different surveyors.

### Electromyographic detection

**Fig. 3:**
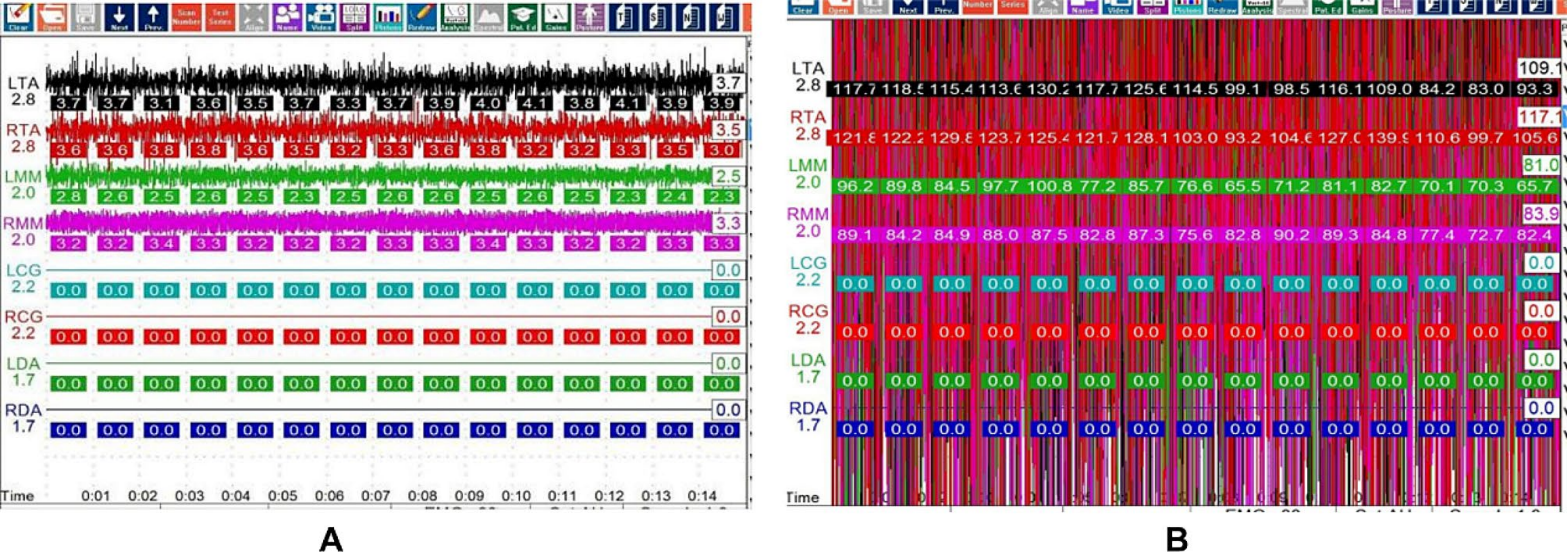
Electromyographic detection at mandibular rest position and teeth clenched position

Electromyogram (EMG) signals of the right and left anterior temporal muscles and masseter were collected and analyzed by the Cranio-Mandibular K7 Evaluation System. In K7 Evaluation System, the measurements from the patient through the eight-channel high quality silver-silver chloride surface electrodes can be read after a pre-amplification gain of 5000 x by EMG Pre-amplifier and passed to the computer via the USB port, then the EMG data were acquired, processed and displayed on the screen by the K7 software. Patient was directed to relax the mandible, data were registered until no visual feedback signals and the EMG reading for all channels were relatively steady on the computer. EMG activities were recorded with mandible at rest position and teeth clenched position. Each tracing needed to complete a full sweep (15 s), and the EMG value were the average of 15 s EMG value. EMG data collection were performed before treatment (T0,) after 6-weeks (T1) and12-weeks (T2) treatment. The side where the mandible molar was recovered was defined as the working side, while the contralateral side as the non-working side. (Fig. 3)

## Results

### Pain scores (NRS) and satisfaction evaluation (5-point likert scale)

During the whole experiment procedure, 22 participants were enrolled into the study based on the eligibility criteria. 4 participants were excluded from further analyses due to be unable to return on schedule. Thus, 18 subjects were included in experimental analysis.

18 subjects completed the treatment plan for a period of 3 months. During treatment, subjects complained of poor pronunciation and the pain of local mucosal or over-erupted maxillary molars although mild (NRS < 4) after the procedure and was similar after the 2-weeks. There was no significant masticatory muscles pain or discomfort in the temporomandibular joint area. All the patients were satisfied with their procedure and treatment effect (Likert scale ≧ 4).

### Intermaxillary vertical space and the teeth transportation distance

**Fig. 4:**
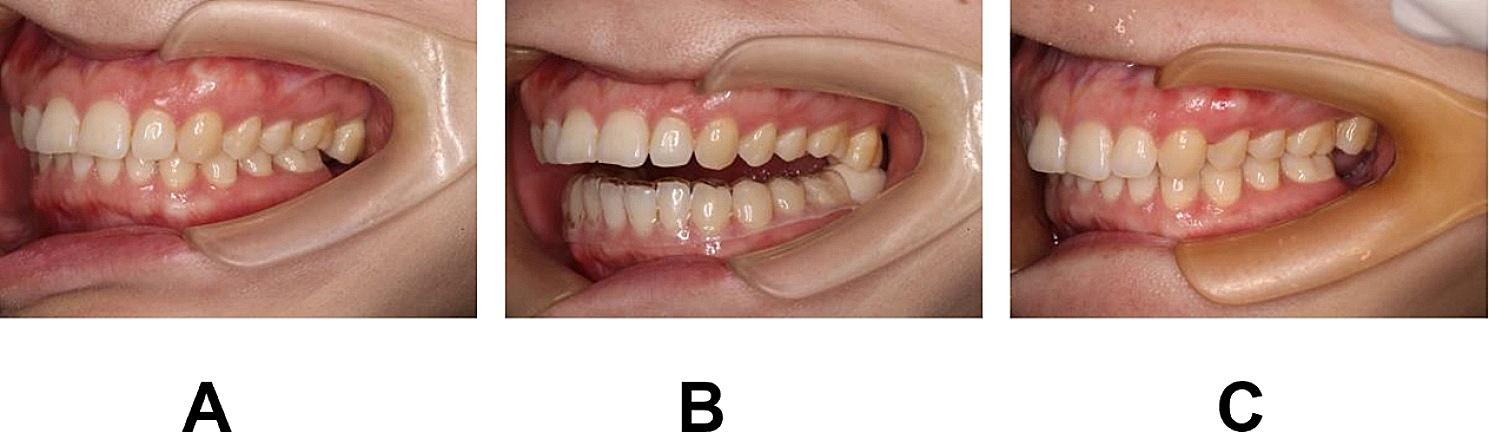
The intermaxillary space in the edentulous area before and after 3 months treatment. **A**, The intermaxillary space in the edentulous area before treatment. **B**, The intermaxillary space in the edentulous area with space-adjustment occlusal splint. **C**, The intermaxillary space in the edentulous area after 3 months treatment

The intermaxillary space in edentulous areas after 6-weeks(T1) or 12-weeks treatment (T2) showed statistically significant increase when compared with the space before treatment(T0) (Fig. 4). On the first 6 weeks, the distance of elongated tip moved was 1.03 ± 0.47 mm, while distance was 0.87 ± 0.33 mm in the next 6 weeks (Table 1).

**Table 1:** Comparison about the data of intermaxillary space at different time points and the teeth transportation distance

		The intermaxillary space		Teeth transportation distance	
	Before treatment (T0)	6 weeks After treatment (T1)	12 weeks After treatment (T2)	T1-T0	T2-T1
Mean ± SD(mm)	1.14 ± 0.86	2.58 ± 0.79^*^	3.73 ± 0.89^#^	1.03 ± 0.47	0.87 ± 0.33

*: EMG values in T1 was compared to that in T0, *P* < 0.05; #:EMG values in T2 was compared to that in T2, *P* < 0.05

### Clinical periodontal parameters

PPT and BI showed no significant difference at 6 weeks after treatment (T1) compared with before treatment (T0). At 12 weeks after treatment (T2), BI showed increasing while PPT have no significant difference. At 2 weeks after treatment ended (T3), PPT and BI showed no significant difference compared with before treatment (T0) (Table 2).

**Table 2:** Periodontal clinical measurements before and after space-adjustment occlusal splint treatment at different time points

	Before treatment(T0)	6 weeks After treatment (T1)	12 weeks After treatment (T2)	2 weeks after treatment ended(T3)	*P*			
	Mean ± SD(mm)	Mean ± SD(mm)	Mean ± SD(mm)	Mean ± SD(mm)	T0/T1	T0/T2	T0/T3	T0/T1/T2/T3
Periodontal Probing depths (PPT)	1.21 ± 0.43	1.41 ± 0.46	1.33 ± 0.48	1.16 ± 0.30	> 0.05	> 0.05	> 0.05	> 0.05
Bleeding index (BI)	0.55 ± 0.51	0.94 ± 0.73	1.22 ± 0.94	0.61 ± 0.50	> 0.05	< 0.05	> 0.05	< 0.05

### Electromyographic detection

EMG data for the masseter muscle in Table 3 and anterior temporal in Table 4 muscles were recorded in both sides before treatment (T0,) after 6-weeks (T1) and12-weeks (T2) treatment. Either on the working side or the non-working side, EMG values showed no statistically significant difference at the rest position and teeth clenched position for the anterior temporal muscles at T1 and T2 compared to T0. No statistically significant differences were found in electromyographic activity of the masseter on the working side at T1 and T2 compared to T0 at mandibular rest position and teeth clenched position. No statistically significant differences were found on the non-working side at T1 compared to T0 at mandibular rest position. While a reduction of muscle activity in masseter on the non-working side were detected at T2 at mandibular rest position.

**Table 3:** Electromyography records of masseter muscles on two sides at different time points

variable	the working side (Mean ± SD)			*P*			the non-working side (Mean ± SD )			*P*		
	T0	T1	T2	T0/T1	T0/T2	T0/T1/T2	T0	T1	T2	T0/T1	T0/T2	T0/T1/T2
resting position	2.21 ± 1.13	1.69 ± 0.98	1.72 ± 0.83	> 0.05	> 0.05	> 0.05	2.22 ± 1.03	1.66 ± 0.83	1.51 ± 0.71	> 0.05	< 0.05	< 0.05
teeth clenched position	122.24 ± 39.61	133.16 ± 46.23	136.37 ± 49.58	> 0.05	> 0.05	> 0.05	126.42 ± 46.82	132.36 ± 40.67	132.49 ± 39.34	> 0.05	> 0.05	> 0.05

**Table 4:** Electromyography records of anterior temporal muscles on two sides at different time points

variable	the working side (Mean ± SD)			*P*			the non-working side (Mean ± SD)			*P*		
	T0	T1	T2	T0/T1	T0/T2	T0/T1/T2	T0	T1	T2	T0/T1	T0/T2	T0/T1/T2
resting position	1.84 ± 0.87	1.80 ± 0.65	1.73 ± 0.74	> 0.05	> 0.05	> 0.05	2.09 ± 1.18	1.97 ± 0.83	1.57 ± 0.75	> 0.05	> 0.05	> 0.05
teeth clenched position	155.58 ± 43.35	149.09 ± 32.98	148.09 ± 36.90	> 0.05	> 0.05	> 0.05	146.51 ± 35.18	146.44 ± 30.07	153.18 ± 33.66	> 0.05	> 0.05	> 0.05

### Statistical analysis

Quantitative data were described and presented by mean plus standard deviation and statistically analyzed by using one-way analyses of variance (ANOVA), which was followed by the least significant difference (LSD-t) multiple comparisons among groups. All statistical analyses were performed with a statistical software program (IBM SPSS Statistics, v25.0; IBM Corp). A p value less than 0.05 was considered statistically significant.

## Discussion

Throughout a person’s life, teeth will continue to erupt through depositing of cementum at the root tip, accompanied by corresponding alveolar bone growth, when the bite force produced by the masticatory muscles counterbalances this budding force, a stable occlusion will be achieved. While the antagonistic tooth is lost, the balanced occlusion is broken, and the teeth continue to erupt due to the continuous deposition of cementum, forming overerupted teeth [16]. Which can lead to insufficient intermaxillary vertical space, great difficulty in prosthetic reconstruction, and cause occlusal interference in protrusive, lateral or retreating movements. The commonest missing teeth in clinic are the first molars and the second molars. In addition, mandibular posterior teeth are more likely to be missing than maxillary posterior teeth [17, 18]. To reconstruct occlusal function in the edentulous area, it is necessary to prepare enough space for dental implant, fixed prosthesis or removable prostheses on the missing mandibular molars. This study was to perform a prospective evaluation of the application of a quite simple, noninvasive approach for treating overerupted maxillary molars in the adult.

In this study, all the participants finished the treatment with great satisfaction about effect of space-adjustment occlusal splint. Local intermaxillary vertical space in edentulous area was significant increased through the application of space-adjustment occlusal splint without affecting the function of the temporalis and masseter muscles. The patient could apply their own occlusal force or chewing pressure to the overeruption maxillary molars, and the overerupted maxillary molar could move in the required direction through adjustment of the occlusal contact points between overeruption maxillary molar and recovered mandible molar in splint. Finally, a relatively normal occlusal curve and sufficient vertical space could be gained for dental implant, fixed prosthesis or removable prostheses.

As a removable device, Splint is often used in the space and stable occlusion maintaining [19, 20], reducing TMD pain and bruxism-related symptoms [21–23], or occlusal reconstruction before orthodontic treatment, surgical treatment or permanent denture restoration [24–28]. Space-adjustment occlusal splint belongs to an improved splint. As a removable device, the medical cost of space-adjustment occlusal splint is significantly lower than that of a fixed appliances ; when you need to attend important banquets or meetings, it can be taken and worn freely with high flexibility; it is fixed and supported through adjacent teeth and corresponding mucous membranes without needing to add another retaining device, which reduces trauma and increases comfort; the retaining device used on tooth is transparent polymer with high aesthetics; at different stages, the occlusal contact point can be adjusted to apply bite force in different directions and adjust the direction of tooth movement. More importantly, space-adjustment occlusal splint is simple to be manufactured and easy to be operated. Most of doctors can solve the problem by themselves, which can reduce the complicated process of referral, effectively shorten the clinical time, enhance the treatment effect, and finally increase patients’ satisfaction.

Occlusion change is one of etiological factors for temporomandibular disorders, which manifests as limitation of mouth opening, muscle pain or joint pain. Pure rotation of the temporomandibular joint aligns with the initial 20–25 mm of incisal opening [29]. Mouth opening movement occurring with no condyle displacement should be considered as a patient-­dependent variable, which realistically increased in case of worn dentition and consequent loss of the original vertical dimension. The available evidence suggested that the stomatognathic system possesses the capacity to swiftly adapt to moderate changes in occlusal vertical dimension [30]. The occlusal height of splint in our study was decided according to intermaxillary vertical distance at mandibular rest position (free-way space). Participants did not experience limitation of mouth opening, muscle pain or joint pain during the treatment process.

To detect whether the elevated occlusal height by splint affecting the functional status of masticatory muscles, the Cranio-Mandibular K7 Evaluation System (MyotronicsNoromed, Inc., Tukwila, WA, USA) was chosen. It is a commercially available, eight-channel EMG device. It can simultaneously monitor eight channels, 4 muscles groups, and can record muscles activity during movement or rest in real time through high quality silver-silver chloride surface electrodes. The information obtained can be amplified, filtered, rectified, digitized and displayed in electromyography on monitor, which can directly reflect the physiological state of the muscles [31–33]. K7 Evaluation System checks electromyographic biofeedback through electrodes adhesive to the surface of the skin, which is convenient to use and does not affect the functional status of the tested muscles. It is suitable for myoelectric activities of the superficial muscles of the maxillofacial region such as masseter, temporalis and digastric muscles. As a painless detection method, it can be used to evaluate the electromyographic characteristics at different time points [34–37]. As an accurate measuring instrument, the EMG signal can be amplified 5000 times and the average emG values measured in 15 s can be displayed on the computer screen, to show the corresponding muscle strength. As an important objective evaluation method after reconstruction, it has been increasingly used in the evaluation of the effect of dental clinical treatment.

The masseter and temporal muscle are symmetrically distributed on both sides of the cranial face, and are the main muscle groups that lift the mandible and make the jaw move closed to produce biting force. Change of force in the masseter and temporal muscles can cause mandible movement disorder and even temporomandibular dysfunction. The masseter and temporal muscle belong to skeletal muscles, which can be triggered to produce an action potential change when excited, and they are located in the superficial layer of the skin, which is easy to detect. In this study, the increase of occlusal vertical dimension with splint for a short time did not affect the electrical activity in the masseter or anterior temporal muscles.

## Conclusions

The results of this study indicates that space-adjustment occlusal splint is an efficient treatment option on overerupted maxillary molars by intruding the maxillary molar to obtain adequate intermaxillary space for prostheses.

## Data Availability

All data generated or analysed during this study were included in this published article.

## Abbreviations

PPT: Periodontal Probing Depth
BI: Bleeding Index
NRS: Numerical Rating Scale
EMG: Electromyogram
ANOVA: One-way Analyses of Variance
LSD-t: The Least Significant Difference
